# Rifaximin for Pertuzumab-Related GI Toxicities

**DOI:** 10.3389/fonc.2017.00168

**Published:** 2017-08-08

**Authors:** Aixa E. Soyano, Gina Reynolds, Alvaro Moreno-Aspitia, Saranya Chumsri

**Affiliations:** ^1^Robert and Monica Jacoby Center for Breast Health, Mayo Clinic, Jacksonville, FL, United States

**Keywords:** HER2 positive breast cancer, rifaximin, pertuzumab, trastuzumab, diarrhea, gastrointestinal toxicities

## Abstract

Pertuzumab is a monoclonal antibody against HER2. Diarrhea and abdominal pain are common adverse events of pertuzumab-based therapy, occurring in almost 70% of patients. The incidence of gastrointestinal toxicities intensifies when pertuzumab is given in combination with chemotherapy. Rifaximin, a non-absorbable oral antibiotic, may provide symptomatic relief in patients with refractory gastrointestinal toxicities from pertuzumab-based therapy beyond standard routine antidiarrheal medications. We present a case of HER2-related therapy-induced diarrhea and abdominal pain managed successfully with Rifaximin.

## Introduction

A 53-year-old woman was diagnosed with stage IIA (pT2 N0 M0) grade 3 invasive ducal carcinoma measuring 3 cm with lymphovascular invasion but negative four resected axillary lymph nodes. Further immunohistochemistry showed that both estrogen and progesterone receptors were focally positive at 10% and HER2 was overexpressed (+3). Since the tumor was larger than 2 cm and HER2 was overexpressed, treatment with adjuvant chemotherapy with TCH + P regimen as described in the TRYPHAENA trial was recommended (1). This regimen included docetaxel 75 mg/m^2^, carboplatin AUC 6, trastuzumab loading dose 8 mg/kg followed by 6 mg/kg every 3 weeks, and pertuzumab 840 mg loading followed by 420 mg every 3 weeks. After the first cycle of TCH + P, she developed severe abdominal pain and cramping (7–8/10 pain scale) and profuse grade 3 watery diarrheas. Despite alternating loperamide and diphenoxylate/atropine, her symptoms continued to worsen, and she had to visit the emergency department. These symptoms, particularly abdominal cramping, continued to worsen during the subsequent cycles of chemotherapy. She was also started on hyoscyamine and probiotics with minimal relief. Given the fact that she did not respond to standard antidiarrheal and antispasmodic therapies, the patient was started on rifaximin 550 mg oral daily for 7 days. Within a few days of initiation of rifaximin, her abdominal pain, cramping, and diarrhea improved. The patient was able to continue and complete her planned six cycles of TCH + P adjuvant chemotherapy without dose reduction or delay. Written informed consent was obtained from the patient for the publication of this case report.

## Background

HER2-directed therapies have revolutionized the treatment outcome for patients with HER2-positive breast cancer (2). One such successful therapy is pertuzumab, a monoclonal antibody that disrupts heterodimerization of HER2 (3). However, gastrointestinal toxicities are common adverse events of pertuzumab. These toxicities can be debilitating and lead to unnecessary dose reduction, treatment interruption, and premature discontinuation, which can result in detrimental outcome. The pharmacologic approaches commonly used to treat pertuzumab-induced gastrointestinal toxicities (PIGT) include general antidiarrheal and antispasmodic agents, such as loperamide, diphenoxylate-atropine, hyoscyamine, etc.

Rifaximin is a non-absorbable oral antibiotic currently approved to be used for irritable bowel syndrome (IBS) with diarrhea (4). We present a case with early stage HER2-positive breast cancer, with PIGT refractory to standard antidiarrheal and antispasmodic agents, who was successfully treated with rifaximin.

## Discussion

Pertuzumab-induced gastrointestinal toxicities are common adverse events of pertuzumab reported in almost 70% of patients receiving pertuzumab, which appear to intensify when pertuzumab is given in combination with chemotherapy. In the pivotal phase III CLEOPATRA trial, which evaluated pertuzumab in combination with trastuzumab and docetaxel in patients with metastatic HER2-positive breast cancer, all-grade diarrhea was reported in 66.8% of patients receiving pertuzumab compared to 46.3% of patients receiving docetaxel and trastuzumab with placebo. Most diarrheas were grade 1 or 2, and the incidence of grade 3 or higher diarrhea only occurred in 7.9% of patients receiving pertuzumab compared to 5% receiving placebo (5). Incidence of all-grade diarrheas was lower after docetaxel was discontinued, with 28.1% in pertuzumab group compared to 14.2% in placebo group (6).

Among patients with early stage HER2-positive breast cancer, all-grade diarrheas have been reported in the range of 43–72.4% in patients receiving pertuzumab combination (1, 7). Incidence of all-grade diarrheas was highest in the TCH + P regimen in the TRYPHAENA trial, 72.4% had all-grade diarrhea and 11.8% of those patients had grade 3 diarrhea or higher. Incidence of all-grade diarrheas in the other two arms was 61.1% (4.2% ≥ grade 3) with 5-fluorouracil, epirubicin, cyclophosphamide (FEC) followed by docetaxel (T), with trastuzumab (H) and pertuzumab (P) given concurrently throughout (FEC + H + P × 3→T + H + P × 3) and 61.3% (5.3% ≥ grade 3) with FEC followed by T + H + P (FEC × 3→T + H + P × 3) arm. Incidence of diarrhea in the phase II NeoSphere trial was similar to that in the TRYPHAENA trial. Incidence of all-grade diarrheas was 51% (7% ≥ grade 3) with pertuzumab, trastuzumab, and docetaxel and 56% (5% ≥ grade 3) with pertuzumab and docetaxel compared to 38% (4% ≥ grade 3) with trastuzumab and docetaxel without pertuzumab. However, incidence of all-grade diarrheas was relatively higher at 43% (3% ≥ grade 3) with pertuzumab and trastuzumab without chemotherapy.

The recent combined analysis of the CLEOPATRA, NeoSphere, and TRYPHAENA trials demonstrated that the incidence of all-grade diarrheas across all studies ranged from 28–72% in the pertuzumab-based treatment arms (8). Most diarrheal episodes were grade 1 (range 21–54%) and grade 2 (range 8–37%). Incidence of grade 3 (range 0–12%) was rarer; there were no grade 4 reported in these 3 trials. Incidence of diarrhea was highest during the first pertuzumab-containing cycle and decreased in subsequent treatments. There was no relationship between pre-existing gastrointestinal comorbidities, such as IBS, colitis, and Crohn’s disease, and diarrheal episodes were observed across all studies. However, only 2% of patients enrolled in these three trials had pre-existing gastrointestinal comorbidities. In the CLEOPATRA trial, incidence of all-grade diarrheas was reported to be similar among patients younger than 65 years vs. those 65 years or older. However, grade 3 or higher diarrhea was higher among patients 65 years old or older (19%) than in patients younger than 65 (8%). Elderly patients were more likely to delay treatment (15% pertuzumab arm vs. 5% control arm) or discontinue treatment (5% pertuzumab arm vs. 1% control arm).

Generally, chemotherapy-induced diarrhea is believed to result from mucosal injury. However, there are several potential mechanisms for diarrhea associated with epidermal growth factor receptor (EGFR)/HER2 signaling blockade, including dysregulation of ion channels in intestinal epithelial cells, which leads to excess chloride secretion; altered gut motility; changes in intestinal microbiome, including small intestinal bacterial overgrowth (SIBO); and altered nutrient metabolism (9, 10). Pharmacologic approaches to treat PIGT are not specific and mainly focus on symptomatic relief. In the previous combined analysis of the CLEOPATRA, NeoSphere, and TRYPHAENA trials, the majority of patients who developed pertuzumab-induced diarrhea were treated with loperamide (8). Besides loperamide, other managements recommended by the National Comprehensive Cancer Network and standard practice guidelines (11, 12) for treating chemotherapy or anti-EGFR-associated diarrhea, are aggressive rehydration, codeine, and stool culture to rule out the possibility of infection. Despite these interventions, dose delays or discontinuations due to diarrhea still occurred in up to 8% of patients receiving pertuzumab. Importantly, dose reductions of pertuzumab were not permitted in the CLEOPATRA, NeoSphere, and TRYPHAENA trials and have not been recommended for treatment of PIGT (8).

Rifaximin is a semisynthetic derivation of rifampin that inhibits bacterial RNA synthesis by binding to β-subunit of bacterial DNA-dependent RNA polymerase (13). Rifaximin inhibits bacterial growth and modulates gut microbiota composition and is currently approved for treatment of IBS with diarrhea (4), hepatic encephalopathy (14), and traveler’s diarrhea. Additionally, rifaximin is being used off-label for *Clostridium difficile-*associated diarrhea. SIBO is a condition in which abnormally large amounts of non-native or native bacteria are present in the small intestine (>10^5^ coliforms/mL) (15). Similar to what has been reported in IBS literature, gastrointestinal problems with diarrhea, flatulence, bloating, and pain in patients receiving cytotoxic chemotherapy are believed to be commonly caused by SIBO (16). However, the mechanisms of PIGT remain largely unknown. Several possible mechanisms have been proposed for EGFR tyrosine kinase inhibitor-induced diarrhea, including negative regulation of chloride secretion causing secretory diarrhea (17), inhibition of EGFR signaling reducing the growth and impairing intestinal epithelium causing mucosal atrophy (18), altered gut motility, colonic crypt damage, changes in the gut microbiome, and altered colonic transport (19). Interestingly, in the Skin Toxicity Evaluation Protocol with Panitumumab trial, prophylactic use of oral antibiotic (doxycycline 100 mg twice daily) to prevent skin toxicity from panitumumab, a monoclonal antibody against EGFR, also showed a significant reduction in all-grade diarrhea (56 vs. 85% with doxycycline) (20). These data support the possibility of SIBO as a mechanism of diarrhea associated with panitumumab.

Based on these data and the fact that PIGT are much more common when given in combination with cytotoxic chemotherapy, we hypothesize that SIBO may be a main mechanism of PIGT. Here, we report a case of a patient with severe, refractory PIGT despite standard antidiarrheal and antispasmodic therapies, who was successfully treated with rifaximin. More importantly, the patient was able to complete her necessary therapy as planned without further dose reduction or delay. Additional studies are needed to evaluate the role of rifaximin in treating or preventing PIGT.

## Concluding Remarks

Pertuzumab-induced gastrointestinal toxicities are common adverse events, affecting up to two-thirds of patients receiving this therapy. The incidence of PIGT is higher when pertuzumab is given in combination with cytotoxic chemotherapy. Despite standard interventions, dose delays or discontinuations due to diarrhea still occurred in a significant number of patients receiving pertuzumab. Studies suggest that SIBO may be one of the mechanisms for PIGT. Rifaximin may provide a treatment option for patients with refractory PIGT. Further studies are needed to determine its safety, tolerability, efficacy, and ability to prevent dose reduction or delay in patients receiving pertuzumab-based chemotherapy.

## Author Contributions

We certify that all individuals listed as authors of this manuscript have participated in conceptualizing the research or content of the manuscript, in writing or critically editing the manuscript, and/or in analysis of data presented in the manuscript.

## Conflict of Interest Statement

The authors declare that the research was conducted in the absence of any commercial or financial relationships that could be construed as a potential conflict of interest.

## Abbreviations

EGFR, epidermal growth factor receptor; FEC, fluorouracil, epirubicin, cyclophosphamide; H, trastuzumab; HER, human epidermal growth factor receptor; IBS, irritable bowel syndrome; P, pertuzumab; SIBO, small intestinal bacterial overgrowth; T, docetaxel.

## References

[1] Schneeweiss A Chia S Hickish T Harvey V Eniu A Hegg R Pertuzumab plus trastuzumab in combination with standard neoadjuvant anthracycline-containing and anthracycline-free chemotherapy regimens in patients with HER2-positive early breast cancer: a randomized phase II cardiac safety study (TRYPHAENA). Ann Oncol (2013) 24(9):2278–84. 10.1093/annonc/mdt182 23704196

[2] Wolff AC Hammond ME Hicks DG Dowsett M McShane LM Allison KH Recommendations for human epidermal growth factor receptor 2 testing in breast cancer: American Society of Clinical Oncology/College of American Pathologists clinical practice guideline update. J Clin Oncol (2013) 31(31):3997–4013. 10.1200/JCO.2013.50.9984 24101045

[3] Al-Hajj M Wicha MS Benito-Hernandez A Morrison SJ Clarke MF Prospective identification of tumorigenic breast cancer cells. Proc Natl Acad Sci U S A (2003) 100(7):3983–8. 10.1073/pnas.0530291100 12629218 PMC153034

[4] Menees SB Maneerattannaporn M Kim HM Chey WD. The efficacy and safety of rifaximin for the irritable bowel syndrome: a systematic review and meta-analysis. Am J Gastroenterol (2012) 107(1):28–35. 10.1038/ajg.2011.355 22045120

[5] Baselga J Cortes J Kim SB Im SA Hegg R Im YH Pertuzumab plus trastuzumab plus docetaxel for metastatic breast cancer. N Engl J Med (2012) 366(2):109–19. 10.1056/NEJMoa1113216 22149875 PMC5705202

[6] Swain SM Baselga J Kim SB Ro J Semiglazov V Campone M Pertuzumab, trastuzumab, and docetaxel in HER2-positive metastatic breast cancer. N Engl J Med (2015) 372(8):724–34. 10.1056/NEJMoa1413513 25693012 PMC5584549

[7] Gianni L Pienkowski T Im YH Roman L Tseng LM Liu MC Efficacy and safety of neoadjuvant pertuzumab and trastuzumab in women with locally advanced, inflammatory, or early HER2-positive breast cancer (NeoSphere): a randomised multicentre, open-label, phase 2 trial. Lancet Oncol (2012) 13(1):25–32. 10.1016/S1470-2045(11)70336-9 22153890

[8] Swain SM Schneeweiss A Gianni L Gao JJ Stein A Waldron-Lynch M Incidence and management of diarrhea in patients with HER2-positive breast cancer treated with pertuzumab. Ann Oncol (2017) 28(4):761–8. 10.1093/annonc/mdw695 28057664 PMC5834072

[9] Agus DB Akita RW Fox WD Lewis GD Higgins B Pisacane PI Targeting ligand-activated ErbB2 signaling inhibits breast and prostate tumor growth. Cancer Cell (2002) 2(2):127–37. 10.1016/S1535-6108(02)00097-1 12204533

[10] Pessi MA Zilembo N Haspinger ER Molino L Di Cosimo S Garassino M Targeted therapy-induced diarrhea: a review of the literature. Crit Rev Oncol Hematol (2014) 90(2):165–79. 10.1016/j.critrevonc.2013.11.008 24373918

[11] Melosky B. Supportive care treatments for toxicities of anti-egfr and other targeted agents. Curr Oncol (2012) 19(Suppl 1):S59–63. 10.3747/co.19.1054 22787412 PMC3377756

[12] Califano R Tariq N Compton S Fitzgerald DA Harwood CA Lal R Expert Consensus on the Management of Adverse Events from EGFR Tyrosine Kinase Inhibitors in the UK. Drugs (2015) 75(12):1335–48. 10.1007/s40265-015-0434-6 26187773 PMC4532717

[13] Hirschmann-Jax C Foster AE Wulf GG Nuchtern JG Jax TW Gobel U A distinct “side population” of cells with high drug efflux capacity in human tumor cells. Proc Natl Acad Sci U S A (2004) 101(39):14228–33. 10.1073/pnas.0400067101 15381773 PMC521140

[14] Bass NM Mullen KD Sanyal A Poordad F Neff G Leevy CB Rifaximin treatment in hepatic encephalopathy. N Engl J Med (2010) 362(12):1071–81. 10.1056/NEJMoa0907893 20335583

[15] Posserud I Stotzer PO Bjornsson ES Abrahamsson H Simren M Small intestinal bacterial overgrowth in patients with irritable bowel syndrome. Gut (2007) 56(6):802–8. 10.1136/gut.2006.108712 17148502 PMC1954873

[16] Andreyev HJ Davidson SE Gillespie C Allum WH Swarbrick E British Society of Gastroenterology Practice guidance on the management of acute and chronic gastrointestinal problems arising as a result of treatment for cancer. Gut (2012) 61(2):179–92. 10.1136/gutjnl-2011-300563 22057051 PMC3245898

[17] Loriot Y Perlemuter G Malka D Penault-Llorca F Boige V Deutsch E Drug insight: gastrointestinal and hepatic adverse effects of molecular-targeted agents in cancer therapy. Nat Clin Pract Oncol (2008) 5(5):268–78. 10.1038/ncponc1087 18349858

[18] Bowen JM. Mechanisms of TKI-induced diarrhea in cancer patients. Curr Opin Support Palliat Care (2013) 7(2):162–7. 10.1097/SPC.0b013e32835ec861 23399616

[19] Yang JC Reguart N Barinoff J Kohler J Uttenreuther-Fischer M Stammberger U Diarrhea associated with afatinib: an oral ErbB family blocker. Expert Rev Anticancer Ther (2013) 13(6):729–36. 10.1586/era.13.31 23506556

[20] Lacouture ME Mitchell EP Piperdi B Pillai MV Shearer H Iannotti N Skin toxicity evaluation protocol with panitumumab (STEPP), a phase II, open-label, randomized trial evaluating the impact of a pre-Emptive Skin treatment regimen on skin toxicities and quality of life in patients with metastatic colorectal cancer. J Clin Oncol (2010) 28(8):1351–7. 10.1200/JCO.2008.21.7828 20142600

